# Soil contamination by *Ancylostoma* spp. and *Toxocara* spp. eggs in elementary school playgrounds in the extreme south of Brazil

**DOI:** 10.1590/S1984-29612022003

**Published:** 2022-01-05

**Authors:** Catia Cilene Santos de Mello, Leandro Quintana Nizoli, Alexsander Ferraz, Bruno Cabral Chagas, William James Domingues Azario, Sara Patron da Motta, Marcos Marreiro Villela

**Affiliations:** 1 Programa de Pós-graduação em Microbiologia e Parasitologia, Instituto de Biologia, Universidade Federal de Pelotas – UFPel, Pelotas, RS, Brasil; 2 Departamento de Veterinária Preventiva, Faculdade de Veterinária, Universidade Federal de Pelotas – UFPel, Pelotas, RS, Brasil; 3 Setor de Saúde Pública Escolar, Secretaria Municipal de Educação e Desporto, Prefeitura Municipal de Pelotas, Pelotas, RS, Brasil; 4 Departamento de Microbiologia e Parasitologia, Instituto de Biologia, Universidade Federal de Pelotas – UFPel, Pelotas, RS, Brasil

**Keywords:** Environmental contamination, larva *migrans*, hookworms, Contaminação ambiental, larva *migrans*, ancilostomídeos

## Abstract

Millions of people worldwide, and especially schoolchildren, may be infected by geohelminths due to their exposure to a contaminated environment. The aim of this study was to evaluate soil contamination by *Ancylostoma* spp. and *Toxocara* spp. eggs in recreation areas at elementary schools in Pelotas, state of Rio Grande do Sul, Brazil. Sand samples were collected from 22 schools and were processed using the centrifugal flotation method. Helminth eggs with zoonotic potential were found in 12 out of the 22 schools (54.5%). Contamination by *Ancylostoma* spp. and *Toxocara* spp. was observed in 36.4% (8/22) and 27.3% (6/22) of the soil samples collected at these schools, respectively. These findings of eggs show that the school communities are exposed to risks of zoonotic transmission.

## Resumo

Milhões de pessoas podem ser acometidas por geohelmintos, especialmente crianças em idade escolar, devido a sua maior exposição a ambientes contaminados. O objetivo deste estudo foi avaliar a contaminação do solo por ovos de *Ancylostoma* spp. e *Toxocara* spp. em áreas de recreação de escolas de ensino fundamental da cidade de Pelotas, Rio Grande do Sul, Brasil. Foram colhidas amostras de areia de 22 escolas e processadas pelo método de centrífugo-flutuação. Em 54,5% (12/22) das escolas houve registro da presença de ovos de helmintos com potencial zoonótico. A contaminação por *Ancylostoma* spp. e *Toxocara* spp. foi observada em 36,4% (8/22) e 27,3% (6/22) das amostras de solo das escolas, respectivamente. Existe a presença de ovos de *Ancylostoma* spp. e *Toxocara* spp., havendo risco de transmissão de zoonoses à comunidade escolar.

Close relationships between humans and pets, such as cats and dogs, are not restricted to their households. Pets also go to public spaces that are used for people’s recreation, and especially places used by children, who are thus exposed to risks of infections spread by some species of helminths that cause diseases (Carvalho & Rocha, 2011).

Since cats and dogs are the domestic animals that have most contact with humans, their sanitary control is essential for human health. Several helminthic diseases that affect pets have zoonotic potential, thus, worm infestations need to be given attention by veterinary care and public health (Woodhall & Fiore, 2014). Humans may get worm diseases when they have direct contact with pets, and through water and food contaminated by eggs or larvae of parasites. Moreover, eggs and larvae may be dispersed in the environment and in pets’ fur, and larvae may actively penetrate through humans’ and pets’ skin. The infections that are transmitted to humans by cats and dogs comprise both cutaneous larva *migrans* (CLM) and visceral/ocular larva *migrans* (VLM/OLM) or toxocariasis and some other diseases (Mendonça et al., 2013).

Human toxocariasis is caused by the migration of *Toxocara* spp. larvae causing clinical and systemic forms (visceral, occult and neurological) and also ocular (MacPherson, 2013). Seroprevalence studies in the southern of Rio Grande do Sul state, Brazil, have found that the seroprevalence of *T*. *canis* in children of the Pelotas, RS, was 50.6%. These authors correlated their finding with the high rate of occurrence of stray dogs in this city (Schoenardie et al., 2013). Furthermore, precautions must also need to be taken about Cutaneos Larva Migrans, may be acquired in the vicinity of schools, given that the larvae of both *Ancylostoma braziliense* and *Ancylostoma caninum* have the capacity to penetrate through the skin, and eggs of these nematodes were detected in feces collected around schools in the county of Pelotas, RS, Brasil (Mello et al., 2020). For these reasons, emphasis should be placed on researching these parasites in the region.

Sandboxes located in public squares and in school playgrounds are very significant for children’s recreation and development. However, it should be highlighted that, since cats and dogs usually have free access to recreation sites, these sites pose potential risks to children who play there (Miranda et al., 2015). When contaminated by helminth eggs and protozoan oocysts that live on animals, these areas constitute an important source of infection for humans (Ghomashlooyan et al., 2015).

It should be noted that schoolchildren comprise a high-risk group regarding geohelminth infections. These individuals are at a stage of life involving much learning, and these kinds of infection have negative impact on cognitive tasks (Mascarenhas & Silva, 2016). Thus, environmental contamination caused by parasites with zoonotic potential, especially at sites where children usually play, such as schoolyards and public squares, has become a relevant issue within public health. Therefore, the aim of this study was to evaluate contamination by helminth eggs with zoonotic potential, in the recreation areas of elementary schools in Pelotas, state of Rio Grande do Sul (RS), Brazil.

**Figure 1:**
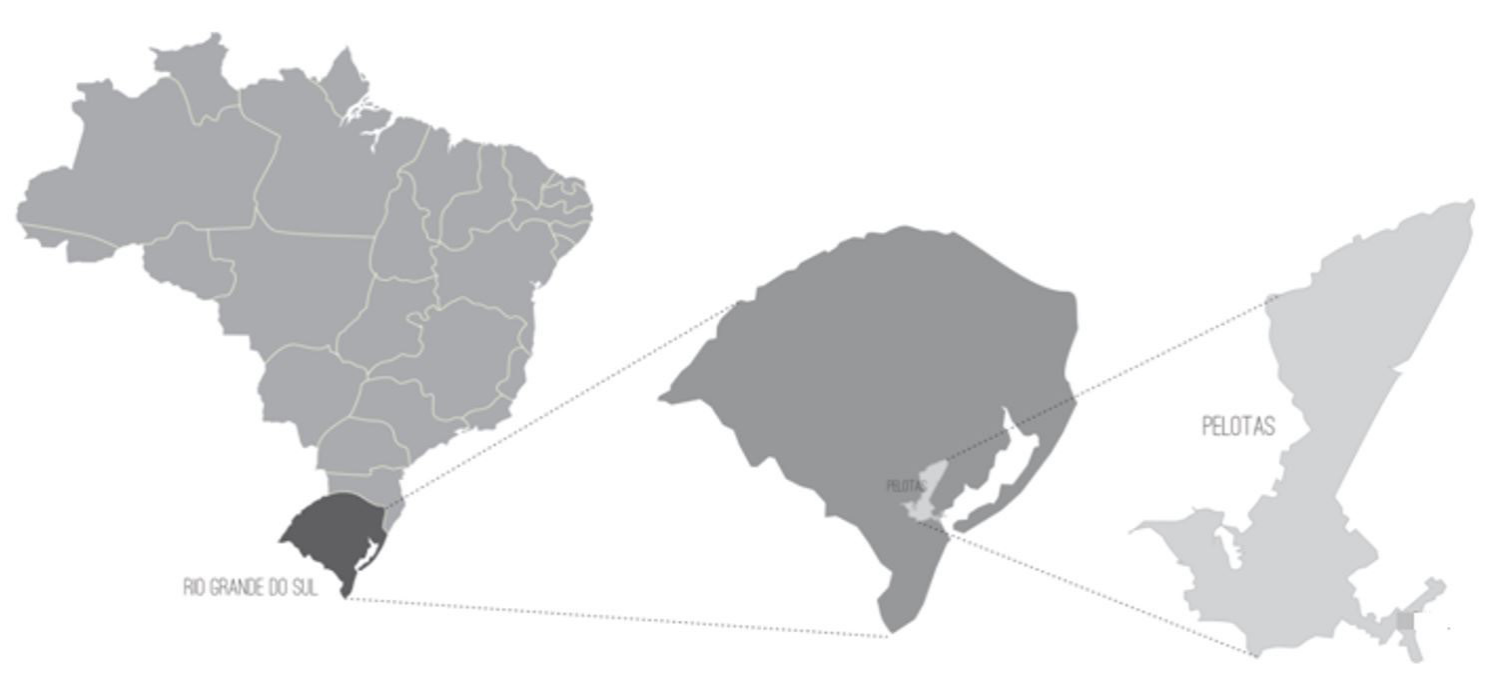
Map of Brazil, Rio Grande do Sul state, southern Brazil, showing the location of the Pelotas.

Meetings between researchers and members of the Municipal Department of Education and Sports in Pelotas, RS, Brazil (Figure 1) led to a partnership that enabled several elementary schools in this city to be selected. They were in four districts and downtown, so that, all quadrants of the city and its central area could be included. A cross-sectional descriptive study was carried out in sand samples collected from 22 schools in the county of Pelotas, between march 2017 and february 2018.

Soil samples were collected from schools in each very quadrant of the city and from schools located in the central area of the municipality. About 250 g of sand was collected from the superficial layer of the soil (not more than 5 cm in depth) with a metal shovel. These were stored as a pool of five samples per school, therefore, resulting in 22 pool samples (Collender et al., 2015). These samples were stored in plastic bags that were appropriately identified. Samples were placed in isothermal boxes and were taken to the Parasitic Diseases Laboratory of the School of Veterinary Sciences, Federal University of Pelotas (UFPel), where they were kept in a refrigerator until the time of analysis. Schools were considered contaminated when at least one of the sites under analysis was positive, since the parasites under investigation have zoonotic potential and may pose risks to schoolchildren.

The sand samples were processed by means of the centrifugal flotation method (Dada & Lindquist, 1979), with some modifications (Ruiz De Ybáñez et al., 2000): 50 g of sand pool were placed a beaker and were homogenized in a 50-ml aqueous solution with mild detergent. Afterwards, the supernatant was transferred to four 10-ml glass tubes, which were then centrifuged (448 G for 5 minutes). The sediments were resuspended in a supersaturated sucrose solution (d=1,230/cm^3^) and the volume of this solution was then made up to form a meniscus on which blades could be placed. Readings were made using an optical microscope (40x magnification) after a 20-minute rest. Eggs were identified by means of morphological and micrometry examination.

The results were expressed as descriptive statistics. The values were described as frequencies (observed value, *n*), arranged in tables and analyzed by means of the Microsoft Excel© software.

Twenty-two schools that were visited over a one-year period had recreation areas with sand. Seventeen were located in districts and five were downtown. Data provided by the Municipal Department of Education and Sports showed that they represented 80.5% of all the schools in Pelotas, RS, with 20,812 students.

The analysis on the sand samples collected at these 22 schools revealed that 54.5% (12) were positive for evolutionary forms of helminths with zoonotic potential. Proportionally, the district with the largest number of positive schools was Areal (80.0%) and the one with the smallest number was Fragata (33.3%) (Table 1).

**Table 1:** Occurrence of contamination by helminth eggs of recreation areas in city schools located in different suburbs in Pelotas, RS, Brazil.

District	Nº of schools	Occurrence	
		N	%
Areal	5	4	80.0
Centro	5	3	60.0
Fragata	6	2	33.3
Laranjal	2	1	50.0
Três Vendas	4	2	50.0
Total	22	12	54.5

*Ancylostoma* spp. and *Toxocara* spp. eggs were detected in 36.4% (8/22) and 27.3% (6/22) of the sand samples, respectively, while eggs of both helminths were found in 9.1% (2/22) of the samples (Table 2). Considering only the positive areas for geohelminths, contamination by *Ancylostoma* spp. and *Toxocara* spp. was observed in 66.7% (8/12) and 50% (6/12) of the contaminated recreation areas, respectively.

**Table 2:** Contamination by helminth eggs of recreation areas in Elementary Schools in Pelotas, RS, Brazil.

Parasite/Association	Frequency	
	N	%
*Ancylostoma* spp.	6	27.3
*Toxocara* spp.	4	18.2
*Ancylostoma* spp. / *Toxocara* spp.	2	9.1
Total positive recreation areas	12	54.5

Parasitic zoonoses in pets have been an emerging threat to public health. This relates not only to changes in epidemiological situations, but also to new studies that have made researchers re-evaluate the risks that are posed by these diseases (Morganx, 2016).

Thus, many investigations into this issue have been carried out worldwide, to warn people of contamination of public squares, parks, schools and their sandboxes. Otero et al. (2018) conducted a study in Lisbon, Portugal, to analyze environmental contamination by *Toxocara* spp. eggs in public parks and playgrounds. Their results showed that 87.5% of playground sandboxes and 50% of public parks were contaminated. Thus, the general prevalence of contamination was 63.2%, i.e. higher than the prevalence found in the present study.

In Bucharest, Romania, the prevalence of positive samples was significantly lower than what was found here, in schoolyards in Pelotas. In that investigation into contamination by canine intestinal parasites, helminth eggs were found in 22% of the soil samples in different parks in Bucharest (Tudor, 2015). This information shows that developed countries worry about this issue, which has an impact on public health. In addition, Kohansal et al. (2017) reported that 19.1% of fecal samples collected in urban areas in Iran were positive for gastrointestinal parasites; 1.8% of the samples contained *Toxocara* spp..

The results from the present study corroborate those of other studies carried out in Brazil, which also showed that eggs and larvae of parasites were prevalent in public recreation areas. This was studied by several authors: Sprenger et al. (2014) found 65% positivity for geohelminths in public areas in Curitiba, PR, where *Ancylostoma* spp. was the most prevalent parasite; and Cirne et al. (2017) evaluated public squares in Valença, RJ, and found contamination by *Ancylostoma* spp. eggs in 66.6% of the sites examined. This last result was very close to our finding of positive schoolyards (66.7%).

In RS, our results showed a higher rate of soil contamination by geohelminths than what was reported in the following studies: Prestes et al. (2015) analyzed 100 soil samples in six cities in southern RS and found 41% positivity; and Moura et al. (2013) observed positivity in 44% (176/400) of soil samples collected from eight public squares in Pelotas, RS. It is noteworthy that for the municipality of Pelotas, it is estimated that there is a population of approximately 100,000 domestic animals, including dogs and cats.

Similar values of environmental contamination by parasites were reported by Chen & Mucci (2012), who found parasitic agents in 50% of daycare centers in Várzea Paulista, SP. While Mascarenhas & da Silva (2016) found that 52% of day care centers in Pelotas, RS, had evolutionary forms of parasites in the soil of their recreation areas. Since that result was very close to the one reported in this paper (54.4%), it suggests that schoolchildren in Pelotas, are at high risk of geohelminth infection and that educational and prophylactic measures are needed. Nevertheless, it is important to emphasize that aspects inherent to the research model can make it difficult to compare different studies (age of animals, investigation site, sample collection model, seasonality, technique used in diagnosis, among other variables).

Dogs were seen in areas inside and outside the schools throughout the time of sample collection, even though most institutions had fences or walls to prevent them from accessing the site. However, according to Marchioro et al. (2013), the most appropriate method for preventing stray dogs from accessing sandboxes is to build fences around recreation areas.

It should be mentioned that these data on school contamination were handed over to the Municipal Department of Education and Sports in Pelotas, RS, and guidelines (printed material) for the students and staff at the schools were recommended.

The contamination rate in the recreation areas of the elementary schools investigated in Pelotas, RS, in the extreme south of Brazil, was high. This may represent an important focus of infection caused by helminths with zoonotic potential and may pose risks to people, and especially to schoolchildren. The findings from this study show the importance of implementing preventive and control measures, such as health education programs, the activity of small animal doctors in the control of potentially zoonotic helminths, use of effective fences, school inspectors to prohibit pets in schools and sandboxes that can be covered at night. Moreover, these measures need to be implemented in association with responsible ownership of pets and control over the numbers of stray dogs.
